# Automating Individualized Formative Feedback in Large Classes Based on a Directed Concept Graph

**DOI:** 10.3389/fpsyg.2017.00260

**Published:** 2017-02-28

**Authors:** Henry E. Schaffer, Karen R. Young, Emily W. Ligon, Diane D. Chapman

**Affiliations:** ^1^Genetics and Office of Information Technology, NC State UniversityRaleigh, NC, USA; ^2^College of Humanities and Social Sciences, NC State UniversityRaleigh, NC, USA; ^3^College of Education, NC State UniversityRaleigh, NC, USA

**Keywords:** automatic assessment tools, formative assessment, instructor interfaces, intelligent tutoring systems, student assessment, learner-content interaction, concept tree, concept graph

## Abstract

Student learning outcomes within courses form the basis for course completion and time-to-graduation statistics, which are of great importance in education, particularly higher education. Budget pressures have led to large classes in which student-to-instructor interaction is very limited. Most of the current efforts to improve student progress in large classes, such as “learning analytics,” (LA) focus on the aspects of student behavior that are found in the logs of Learning Management Systems (LMS), for example, frequency of signing in, time spent on each page, and grades. These are important, but are distant from providing help to the student making insufficient progress in a course. We describe a computer analytical methodology which includes a dissection of the concepts in the course, expressed as a directed graph, that are applied to test questions, and uses performance on these questions to provide formative feedback to each student in any course format: face-to-face, blended, flipped, or online. Each student receives individualized assistance in a scalable and affordable manner. It works with any class delivery technology, textbook, and learning management system.

## Introduction/motivation for project

An essential component of instruction is paying attention to and supporting the learning needs of individual students both in face-to-face and in online environments. While instructors develop the course for the benefit of the class as a whole prior to its start, fine-tuning of instruction while the course is ongoing must take place for each individual student to reach his or her maximum learning potential. However, fine-tuning instruction for individual students may not be feasible in a large class. When teaching a class of up to 15 students, the instructor can interact with each one individually every class meeting. Instructors of such courses are able to give immediate feedback concerning missed or misunderstood subject matter as well as provide an overview of progress and barriers to that progress. For larger classes, up to perhaps 35 students, this interaction may happen about once a week. For even larger classes, there is little hope of student-instructor interaction unless a student seeks out the instructor (Cuseo, 2007). This lack of interaction may also be true even for smaller online classes. The students who most need help are often the least likely to go to see the instructor. This is disappointing for an instructor who is concerned with, and indeed motivated by, positive student learning outcomes. While students in higher education do have an increased responsibility for monitoring their learning and seeking help, they encounter these larger classes more frequently in their early years and formative feedback may assist the development of learner autonomy. While this clearly falls in the area of learner-content interaction (Moore, 1989), we consider this to fall more specifically into the category of instructor-guided learner-content interaction. It fits into this category because of its one-way communication.

Class sizes in the hundreds are common in many large institutions of higher education and can reach over a thousand. The largest massively open online courses (MOOCs) have had enrollments over 100,000. There have been many efforts to identify strategies to offer sufficient personalized attention to individual students in large classes and an increasingly popular approach is the use of peer grading (Bradley, 2013; Duhring, 2013; Piech et al., 2013; Shah et al., 2013; NC State University, 2014), which has prompted both negative reviews (e.g., Rees, 2013) and mixed negative/positive reviews (e.g., Luo and Robinson, 2014). Overall course completion rates (Fiedor, 2007; EdX, 2014; Kolowich, 2014) have been studied with an emphasis on such areas as grades, persistence, motivation, and readiness. Yet low course completion rates continue to be a problem leading to increased time to graduation and failure to graduate. Low retention and graduation rates are affected by many factors, which differ greatly in nature, and are serious problems for an institution. They originate with insufficient student progress toward learning outcomes within individual courses and must be addressed at that level. This project focuses within a course to support individual student progress in achieving course learning outcomes.

## Related works/state of the art

An overview of four categories of approaches to analytical activities that are currently being used on data from educational settings is provided by Piety et al. (2014). Their work provides a conceptual framework for considering these different approaches and provides an overview of the state of the art in each of the four categories. Our work falls primarily into their second category, “Learning Analytics/Educational Data Mining.” Their work identifies the areas of overlap between their four different categories and a noticeable gap is left by the current approaches in the educational context of individual students in postsecondary education. This gap is the focal area for our current work and what follows is a description of the state of the art in the Learning Analytics category as it relates to our work.

### Log based approaches

Much attention has been paid to using information from Learning Management Systems (LMS) logs and other logs of student activity. These logs are used to flag students who are likely to do poorly in a course and/or make satisfactory progress toward graduation. A survey article in the *Chronicle of Higher Education* (Blumenstyk, 2014) describes this as “personalized education” but considers the term to be “rather fuzzy.” This area is also often referred to as “learning analytics” (LA). Many tools have been developed to help colleges and universities spot students who are more likely to fail (Blumenstyk, 2014; Rogers et al., 2014). Companies with offerings in this area include Blackboard^1^, Ellucian^2^, Starfish Retention Solutions^3^, and GradesFirst^4^. The details of what data these companies use is not clear from their web sites, but their services generally appear to utilize LMS logs, gradebooks, number and time of meetings with tutors and other behavioral information, as well as student grades in previous courses. Dell has partnered with a number of higher education institutions to apply this type of analytics to increase student engagement and retention, such as at Harper College (Dell Inc, 2014). Their model emphasizes pre-enrollment information, such as high school GPA and current employment status. These efforts often produce insight into progress of the student body as a whole, and to individual students' progress over the semesters, but do not go deeper into individual student's learning progress within a course.

### Approaches based on student decisions

Civitas Learning^5^ takes a different approach. It emphasizes the need to inform the student regarding the decisions to be made in choosing the school, the major, the career goals, the courses within the school, etc. These are very important decisions, and certainly can be informed by a “predictive analytics platform,” but they are outside an individual course. Ellucian^6^ describes their “student success” software in much the same way, but in less detail. Starfish Retention Solutions^7^ also describes its software in much the same way and gathers data from a variety of campus data sources, including the student information system and the learning management system. The orientation, as described, is at the macroscopic level, outside of individual courses. An example given is that when a student fails to choose a major on time, an intervention should be scheduled to assist in student retention. GradesFirst^8^ describes its analytics capabilities in terms of tracking individual student course attendance, scheduling tutoring appointments, as well as other time and behavior management functions.

### Course concept based approaches

Products and services from another group of companies promote the achievement of student learning outcomes within courses by adapting the presentation of material in the subject matter to the progress and behavior of individual students. This is sometimes referred to as ”adaptive education” or “adaptive learning.” One company, Acrobatiq^9^, distinguishes between the usual learning analytics and their own approach (Hampson, 2014) and does it in the domain of an online course specifically developed to provide immediate feedback to students. This is an interesting and promising method, but its application appears to be limited by the need to develop a new course, rather than being directly applicable to existing courses.

Smart Sparrow^10^ describes its function as “adaptive learning,” looking at problem areas encountered by each student and personalizing the instructional content for each individual student. The company describes this in terms of having the instructor develop content using their authoring tool, which then allows presentation of the next “page” to be based on previous student responses. This appears to be a modern instantiation of Programmed Instruction (Radcliffe, 2007).

WebAssign^11^ is a popular tool used in math and sciences for administering quizzes, homework, practice exercises, and other assessment instruments. Their new Class Insights product appears to provide instructors with the ability to identify questions and topic areas that are challenging to individual students as well as the class collectively (Benien, 2015). It also provides feedback to students to help them identify ways to redirect their efforts if they are struggling to generate correct answers to questions and problems. Aplia^12^ provides automated grading services for instructors with feedback intended to help students increase their level of engagement. They create study plans for students based on how they performed on their quizzes, which are created using a scaffolded learning path moving students from lower order thinking skills to higher order thinking skills. These plans are not shared with the instructors and are for students only.

Textbook publishers have been developing technology solutions to enhance their product offerings. CengageNow^13^ has pre and post assessments for chapters that create a personalized study plan for students linked to videos and chapters within the book. Other textbook publishers have a similar approach in their technologies. In contrast, the Cengage MindTap^14^ platform has an engagement tracker that flags students who are not performing well in the class on quizzes and interaction. This is more focused on providing the instructor with information to intervene. A dozen or so student behaviors and interactions are used to calculate an engagement score for each student in MindTap, including student-generated materials within the content. McGraw Hill also offers adaptive learning technology called LearnSmart^15^ which focuses on determining students' knowledge and strength areas and adapts content to help students focus their learning efforts on material they do not already know. It provides reports for both instructors and students to keep updated on a student's progress in a course.

This adaptive learning approach, along with methods to select the path the student should take from one course content segment to the next, is used by many implementations of Adaptive Educational Systems. An example is the Mobile Integrated and Individualized Course (MIIC) system (Brinton et al., 2015), a full presentation platform which includes text, videos, quizzes, and its own social learning network. It is based on a back-end web server and client-device-side software installed on the student's tablet computer. The tests of MIIC used a textbook written by the implementers and so avoided permission concerns. Another service, WileyPLUS with ORION Wiley^16^, is currently available with two psychology textbooks published by the Wiley textbook company. It appears to use logs and quizzes, along with online access to the textbooks, in following student progress and difficulties. It seems to be the LMS for a single Wiley course/textbook. In this case, there is no development by the instructor needed, but one is limited to the textbooks and approach of this publisher.

### Shortcomings/limitations of current approaches

What the varied approaches in the first two categories (Log and Student Decision Based Approaches) apparently do not do constitutes a significant omission; the approaches do not provide assistance to students with learning the content within each course. While informing students can improve their decisions, the approaches described in Student Decision Based Approaches impact a macro level of student decision making; the project described here relates to student decision making at a micro level. Providing individual face-to-face support within a course is time-consuming, which makes doing so expensive. The increasing number of large courses is financially driven, so any solution to improve student learning must be cost effective. Cost is a major limitation of the approaches described in Course Concept Based Approaches. With those approaches, existing instructional content must be adapted to the system, or new instructional content must be developed, essentially constructing a new textbook for the course. That is not a viable option for most individual instructors, forcing them to rely upon the content developed by someone else, such as a textbook publisher. Often, instructors find some aspects of their textbook unsatisfying and it may be difficult to make modifications when a textbook is integrated within a publisher's software system. The tool proposed in this paper avoids that problem.

## The proposed tool

In light of the shortcomings previously mentioned, we developed a software tool that would assist instructors in achieving the goal of improving student learning without limiting their intellectual or pedagogical freedom or imposing prohibitive time or service costs. It has three elements: it tracks individual student performance, it provides the instructor with item analysis data, and for each individual student, it provides formative assessment based on the course concepts.

### Tracking individual student performance

We start with the first dimension, that of tracking individual performance on tests and assignments. In today's LMS, quiz or exam results (summative assessments) are communicated to students, instructors, and sometimes to academic advisors, but provide only very minimal help. A low grade means the student performed poorly, but the score itself tells the student nothing about where the problems were experienced or how to develop a better understanding of the material. When students review their individual test questions to determine the errors they made, that provides only minimal assistance in understanding where their knowledge gaps lie because analyzing the missed questions requires the student to have deep understanding in the areas where a lack of understanding has been demonstrated. Students typically need feedback from the instructor or teaching assistant to develop an understanding of the knowledge gaps. However, in large classes, there may be too many students for instructors and teaching assistants to provide such support for all struggling students. The students who are not struggling, but who have problems with particular aspects of the course material, are even more numerous. Therefore, new methods of providing such feedback to students in a cost-effective manner are needed. Computer-assisted methods may be able to bridge this gap. Given that most large courses utilize quizzes and exams comprised of objective questions (e.g., multiple choice or true/false items) that can be easily graded by a machine and usually are, our initial work uses such assessments. Other researchers working in the learning analytics space have been developing tools to support formative assessment of students' written responses to short answer questions (e.g., see Leeman-Munk et al., 2014).

### Item analysis and quality assessment

Computers have been used for many years for analyzing exam results. One of the older computer-assisted methods for improving testing is item analysis. The proposed tool, adds to the benefits of item analysis by providing formative feedback to students. It will be briefly covered below, as it should be included as part of an overall teaching and assessment strategy. It is usually considered to be in the area of psychometric analysis and is standard content for any course and textbook on psychological testing. While item analysis originated in the disciplines of psychology and education, and is very broadly known and utilized there, it is generally not utilized in instruction in most STEM disciplines which rely heavily upon objective questions.

While item analysis first focuses on items (individual questions), a very important early addition provides a view of the test as a whole. This analysis of internal test reliability (Kuder and Richardson, 1937) (commonly known as KR20) indicates how well the exam, taken as a whole, distinguishes between students with varying mastery of the material. Similarly, there are conceptually-related measures, including point-biserial correlation and the discrimination index, which indicate for each question whether the students with a higher grade tend to answer it correctly more than do students with lower grades. This provides a measure of question quality that reveals inadvertent “trick” questions. It is easy, unfortunately, for a question intended to be straightforward, to be inadvertently phrased in a manner which misdirects students who have a deeper understanding of the subject to a distractor answer while allowing students with a shallow understanding to arrive at the correct answer. Analyzing exams and using such feedback to improve question construction allows tests to more accurately assess student learning.

High-quality exams are important in assessing student progress. Two separate goals of assessment, summative and formative (Scriven, 1967), are too often conflated. Summative assessment is conducted to determine whether or not students have achieved the level of knowledge and skills expected at the end of a course module and are typically used to determine grades. However, summative assessment provides little to no help to students in mastering the material within a course. Formative assessment is conducted to observe students' progress and provide feedback that assists them in achieving educational objectives. These may or may not be graded. Given that educational institutions have a mission to develop students, which clearly includes mastering the material within courses, formative assessment is fundamental to supporting this mission.

### Inputs for formative feedback from assessment

The core of the method described here is the analysis of student responses to test questions in conjunction with specific subject matter data (“metadata” for each test item), which is used to generate diagnostic feedback to students individually. This cannot be done in the usual grading context where the only information available for analysis is the correct answer and the resulting grade. Providing metadata is required to allow useful analysis, but this adds a significant cost to the test-development process. In the methodology proposed here, the cost of developing this metadata is a “fixed cost” for the class, with a zero “marginal cost” resulting from enrolling additional students into the class. This results in significant economies of scale, and so fits well into a large-class scenario in which marginal cost is an extremely important consideration.

**Figure 1:**
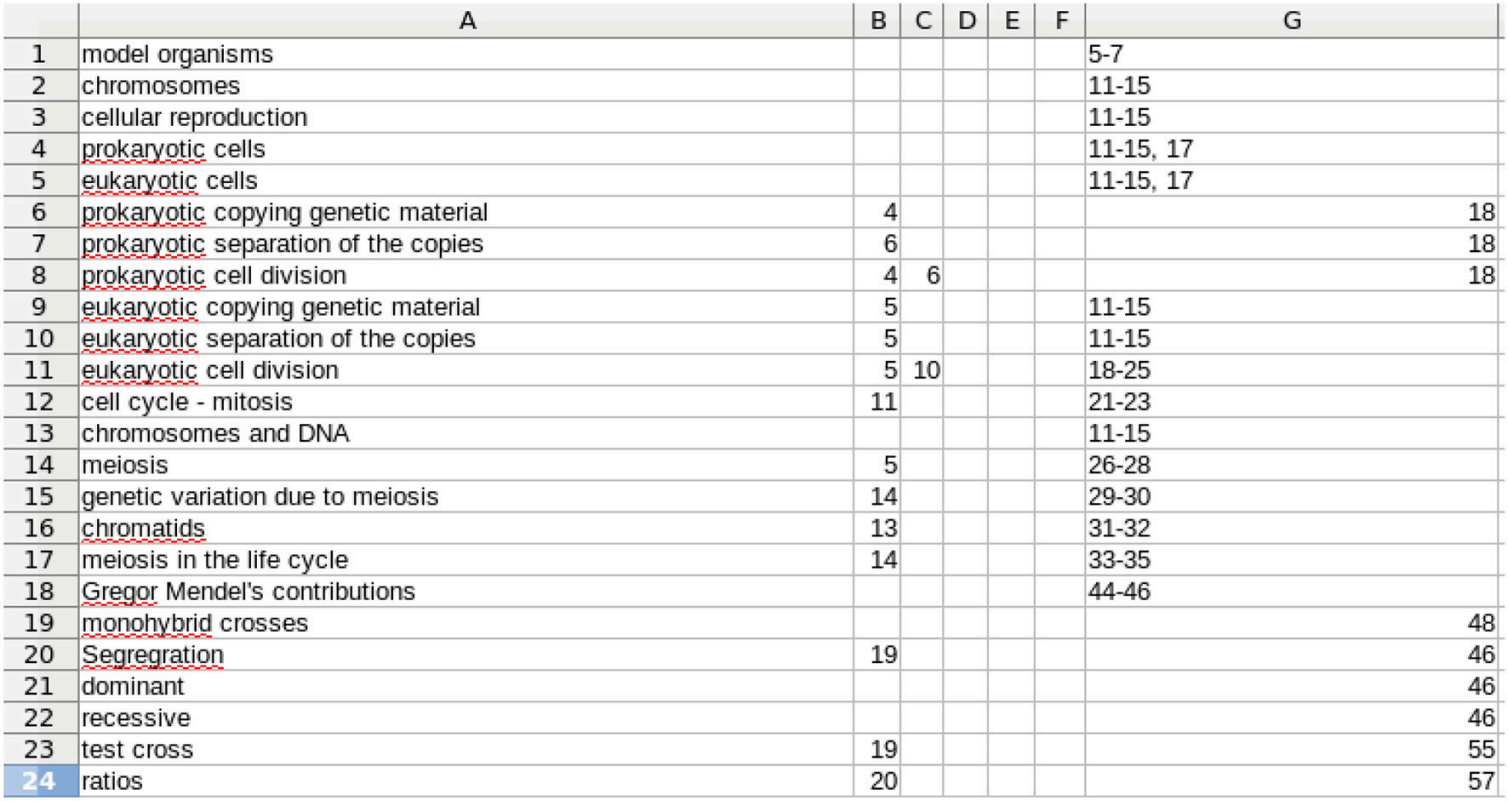
**A screen capture of a portion of a Concept Table for an introductory genetics course**. Column A gives the concept to be learned. Columns B–F are up to 5 IPCs. The limit of 5 is arbitrary and was chosen because that was all that was needed for the two courses discussed here. Note that the entries in Columns B–F are row numbers. Column G is free text and identifies resources that support learning of that row's concept. It typically contains the relevant page numbers in the textbook, links to relevant on-line material, etc. The full table continues the same scheme with additional rows, 115 in total for this particular course.

**Figure 2:**
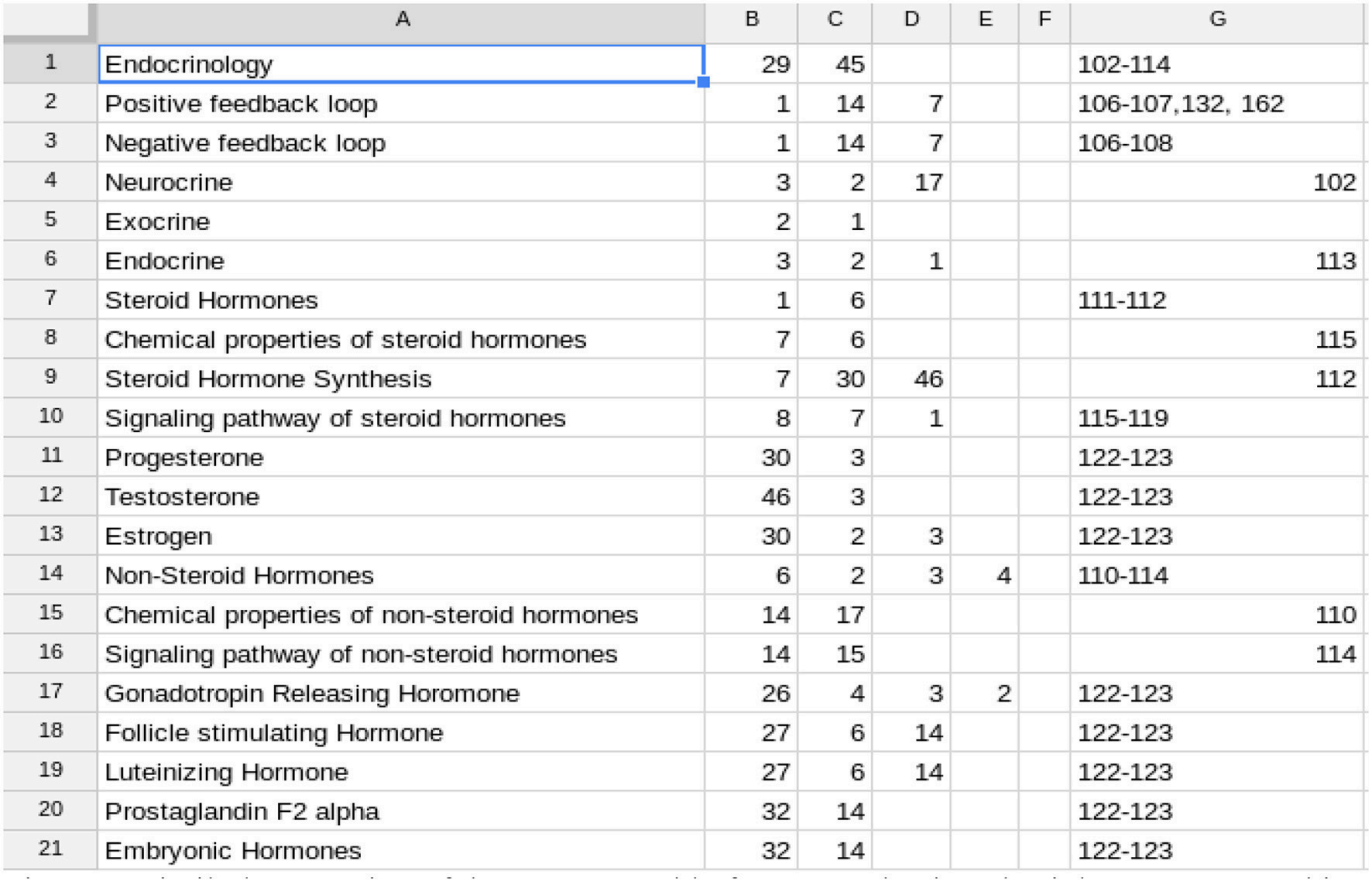
**Similarly, a portion of the Concept Table for a reproductive physiology course**. In this course, there were 75 rows in the complete table. In this Table the IPCs (columns B–F) often refer to rows below the one in which they are mentioned. This is because the concepts in the table are not listed in the order in which they are introduced in the course. This does not affect the students as they use the alphabetically arranged listing. See Figure 7.

Application of the proposed tool starts with the enumeration of the concepts and capabilities that each student must develop and master in the course. There are two types of metadata to be developed. The first relates to the concepts and the concept tree, which form the structure of the course. This concept metadata must be produced by the instructor, as it requires expertise in the subject matter, experience in teaching the subject, and knowledge of the structure and coverage in the specific course. Assuming this level of experience and expertise, developing the metadata requires a moderate amount of effort. A review of the textbook(s) and other resources is needed, as well as the class lecture material and reflection on the key concepts and the supporting concepts (Immediate Predecessor Concepts or IPCs in our terminology) on which each concept is based. Additionally, the course resources, such as pages in the text covering each concept, are included in this metadata and will be provided to the students later as the analysis directs. A quick first view of the concepts can be obtained by reviewing the detailed Table of Contents of the adopted textbook, especially when the course follows the textbook reasonably well. However, the full array of concepts and IPCs (often referred to as a concept tree, but in actuality a directed graph) is not fully specified by the Table of Contents. Rather, it takes expertise in the subject to select the concepts and the IPCs which underlie each specific concept. In our experience, producing this metadata represents an investment of 8–12 h of thoughtful first-time development for a typical three to four credit course. See Figures 1, 2 for examples of the Concept Table in two different courses.

The format illustrated by Figure 1 was deliberately chosen because it (a) is easier and faster for the instructor to enter a row number than to type in the full name of each IPC, (b) makes the table more compact and so easier to view and work with, and (c) eliminates typographical errors in entry of IPC names, which is important for the computer processing described below. Row order is unimportant. In Figure 1, the row order follows the chronological flow of the concepts taught in the course. Modification of row order is discussed in the following section.

Development of the Concept Table is a one-time, up-front effort for a class, with the effort independent of class size. It will need to be reviewed each time the course is taught, but is likely to remain substantially the same. Therefore, the fixed cost can be much lower for subsequent classes. The faculty member who developed the Concept Table in Figure 2 kept track of his time and spent 7¾ h on this. In the next semester, it took 1 h to make minor adjustments.

If the textbook is changed, there will be additional effort required to change the listed resources, but again, this is a one-time effort. As a result, the effort required will be lower in subsequent classes. The format of the Concept Table shown in Figures 1, 2 is chosen to make it easy for the instructor to populate and for the analysis program to use. However, this format does not allow for easy major revision. To facilitate more substantial revision, including deleting, adding, and rearranging rows, we have a computer program (developed for this project) which fills in the full names of the IPCs in columns B–F. Then rows can be rearranged and we have another program to return to the abbreviated version. This minimizes the effort needed to produce and revise the Concept Table.

This easing of revision is relevant to economy of scale, as the effort needed to produce the Concept Table for one instructor's class can then be applied to a course taught in many different sections by many different instructors. In such a case, each instructor can use it as is or revise it for that specific instructor's use. In one case in our experience, when the same textbook was adopted, the second instructor needed <1 h to revise the first instructor's Concept Table. This provides additional scalability. Continuing along economies of scale, the Concept Table could also be produced by the publisher to accompany the textbook, providing the instructor a basis from which to start. All of these lead to a decrease in the up-front (fixed) investment. From that start, the marginal cost increase for larger and larger courses is zero.

**Figure 3:**
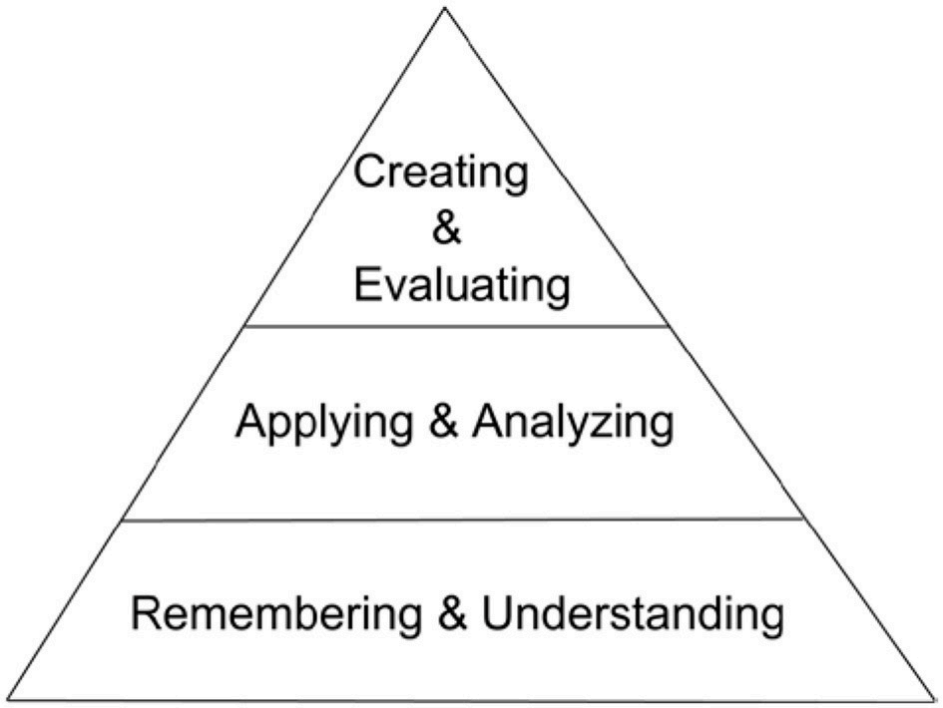
**A simplified revised Bloom's Taxonomy of Educational Objectives**.

The second part of the course metadata requires one more significant fixed effort. This is the annotation of every test question to be administered to the students, and so can be done in pieces before each test. In addition to indicating the concept(s) tested, this annotation process includes the instructor's evaluation of the level of cognitive demand the question places on the student with regards to a modified version of Bloom's Taxonomy (Bloom, 1956). Bloom's Taxonomy is a tiered model of classifying thinking into levels of cognitive complexity. The original taxonomy included six levels going from requiring the least cognitive complexity to the most (knowledge, comprehension, application, analysis, synthesis, and evaluation). Anderson and Krathwohl (2001) proposed a revised taxonomy with the noticeable differences being the replacement of the synthesis level with that of creating and moving creating to the top level. The revised taxonomy, shown in the condensed array we use, is represented visually in Figure 3.

The revised taxonomy of educational objectives (Remembering -> Understanding -> Applying -> Analyzing -> Evaluating–Creating) is a very thorough classification system of a complex area of study in educational theory. While our work with the taxonomy is focused at the concept- and content-level of a course, it can also be applied to a course-level analysis within the learning analytics space (Gibson et al., 2014). It can be difficult for experts to determine exactly which level is being assessed by an individual test question and that level of granularity may not be useful to an instructor and/or the students. We have found that a condensation of Bloom's Taxonomy into three levels makes it much easier for students to interpret and apply to their studying. Some fine distinctions are lost, but the qualitative distinctions between the newly grouped levels remain and are satisfactory for identifying student challenges related to insufficient progress. We call the Remembering and Understanding level Low (L), the Applying and Analyzing level Medium (M), and the Evaluating, and Creating level High (H) as shown in Figure 3. The time needed for a question testing one concept at a Bloom's L level is perhaps 1 min in addition to composing the question. For a more complex question, 3–5 min may be needed. For reworked questions based on previously annotated questions, 1–2 min should suffice.

**Figure 4:**
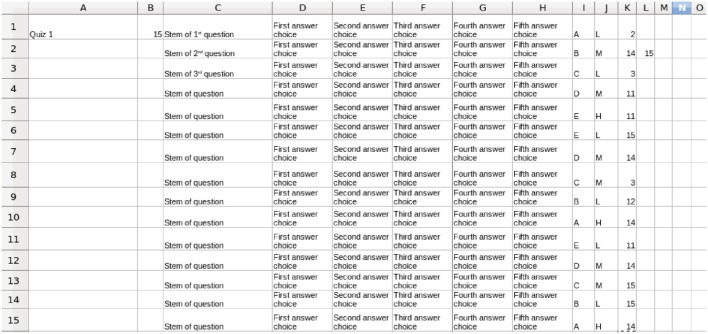
**A schematic example of a quiz annotated with metadata**. For each question, the correct answer is in column I, the Bloom's Level is in column J, and up to 5 concepts being tested in each question are listed in columns K–O. The contents of columns K–O refer to the rows of the Concept Table for the course shown in Figure 1. The stems and answer choices are shown here generically, and are not be used in the processing described later in this paper.

Figure 4 shows a schematic example input quiz consisting of multiple-choice questions tagged with the metadata described above. Any objective question can lend itself to this type of analysis. The cognitive level metadata must be constructed for each course because the level depends not only on the question, but also on the prior learning situation of the students in the course. For example, students who have had practice with applying and analyzing the information in a specific scenario might be answering a related question simply by remembering previous discussions from class. While a test item might appear to be testing at a higher level, these students would actually be performing the most basic level, recall of knowledge. However, a student taking the same course with a different instructor who does not use that example scenario would be tested at a higher cognitive level by that same test item.

### Outputs of the tool

**Figure 5:**
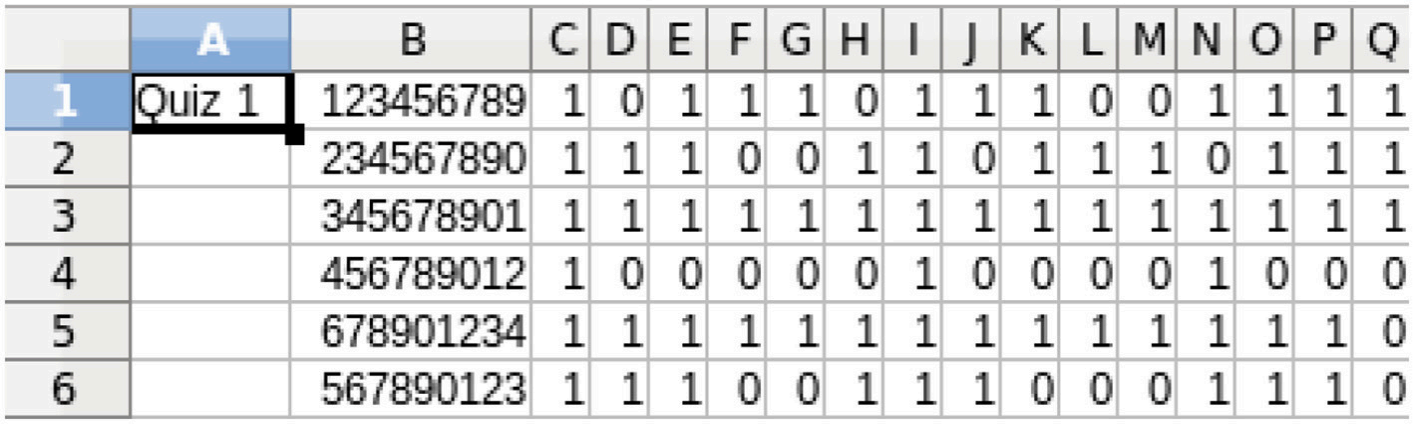
**Student quiz results for the 15-item quiz in Figure **4****. 1 = correct, 0 = incorrect. The student IDs are in column B. These results are downloaded from an LMS or obtained from optical scanning and, therefore, can be in formats other than shown.

**Figure 6:**
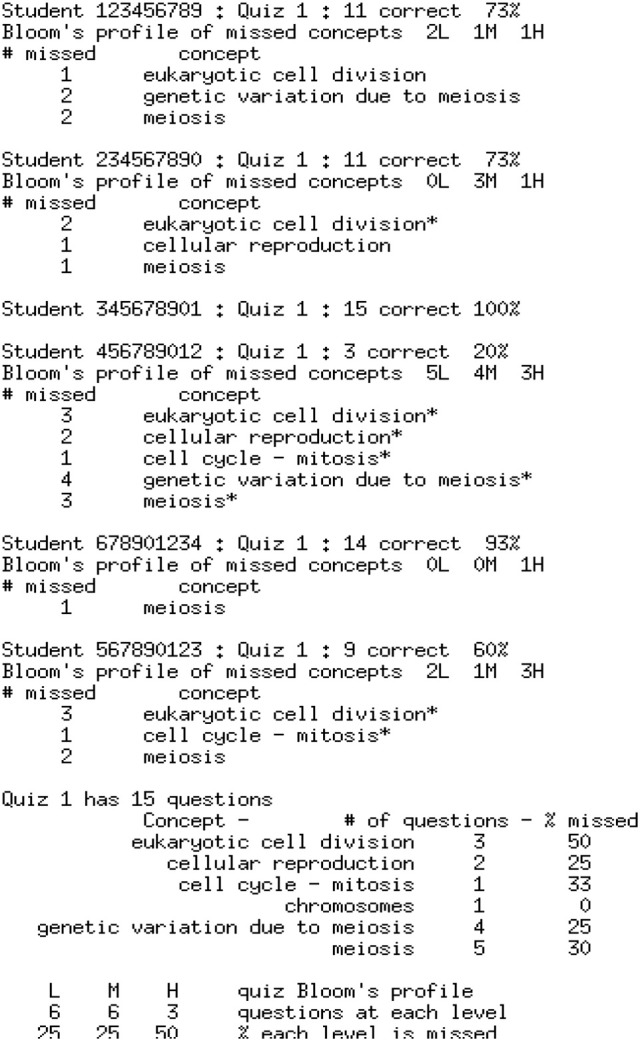
**The report of each student's performance, as provided to the instructor**. The questions answered incorrectly by each student have their tested concepts listed, with an asterisk flagging those concepts answered incorrectly over 50% of the time. (This value can easily be adjusted). At the end is the summary provided only to the instructor.

The two metadata sources described in Section Inputs for Formative Feedback from Assessment provide the background material for the analysis of the quiz results. Figure 5 shows an example of the quiz results for a class of six students who have completed a 15-item quiz. The quiz results are analyzed using a program developed in this project based on the metadata contained in Figures 1, 4 and a report is prepared for each student. The individual student reports can be distributed electronically in whatever manner is most effective for the students and instructor(s); we have used email. The student reports, plus a class summary, are provided to the instructor, as shown in Figure 6.

**Figure 7:**
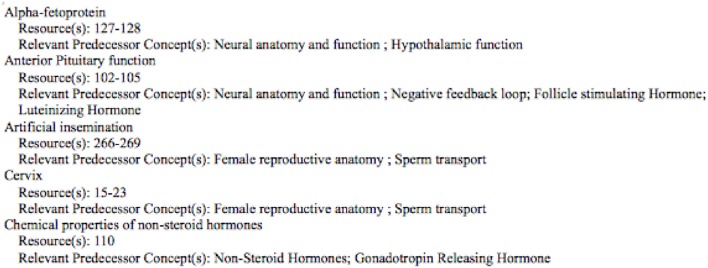
**The student version of the Concept Table in Figure **2****. Only the initial portion of the concept listing is shown.

Each student receives his/her individual test report, the appropriate subsection of the larger report shown in Figure 6, which gives a summary of concepts missed and a Bloom's profile of the cognitive level of questions missed. Students also need information and guidance on how to fill in the knowledge gaps identified by the test report. One way to do this is to provide students with a student-friendly list (Figure 7) of the information from the Concept Table in Figure 2, created via a program developed for this project. Another option is to embed the information related to incorrect test items in the individual student report. While students may prefer this option, it has two clear disadvantages. It generates a much longer instructor report and it prompts students to review only missed information. A full Concept Table, such as shown in Figure 7, may promote student awareness of the interconnectedness of a greater number of concepts and may prompt review of other tested concepts, in addition to those missed by the student on the specific exam.

Each student report only traces back to the immediate predecessor concepts. Students who are still struggling with previous IPCs from earlier units/quizzes may need to consult previous test reports and track back IPCs as shown in Figure 7 in order to achieve the level of understanding and mastery necessary for progress in the course. In an interactive LMS or other computer-supported, learning environment, a student can be offered additional quizzing on the concept(s) missed, with or without the possibility of earning back a fraction of the points missed earlier.

In addition to reviewing the concepts, it should be taken into account that test items on a given concept can be formulated to test at different cognitive levels. Therefore, an additional aspect of student support is to help them understand the Bloom's profile provided by the report, so that they may utilize it in their studying. In our experience, Bloom's Taxonomy of Educational Objectives is not well understood by most instructors in fields other than Education and Psychology. Even fewer students have ever heard of this classification. A relatively small amount of class time must be devoted to explaining this area and the instructor should provide material to support the explanation. Our pilot test results indicate a substantial, but minority, student interest in learning to interpret Bloom's Taxonomy and applying it to help them to do a better job studying and responding to test questions. This supports the value of introducing Bloom's Taxonomy to the students (and the instructor, if necessary). There are many introductory resources available on the web.

With all of the above material, a student wishing to improve performance in the subject matter has the tools needed to fill in areas of weakness. However, the analyses described above also assist the faculty member. The faculty report, shown at the bottom of Figure 6, provides the analysis of overall class performance. This report is substantively different than a graph of the frequency count of test scores, as it shows outcomes both in terms of success in mastering concepts as well as a profile of accomplishment in terms of Bloom's level. The faculty member can thereby tailor instruction in response to class performance and needs. This can assist in balancing course content with respect to Bloom's level. The modification of course content will vary for each class section, so quiz by quiz feedback is essential to providing insight into how to tailor the class to those specific students. This type of assessment relies heavily on test item quality, even more than does simple assignment of grades, increasing the importance of item analysis. It provides a basis for the instructor to review test items in order to improve or even remove them. It promotes a focus on assessing concepts and supports the removal and avoidance of poorly stated questions.

### Costs for tool implementation

All of this reporting on the progress of student learning relies on the computer program developed for this project, which generates the output shown in Figure 6. Its development represented a substantial investment in planning, specifications development, program development, program testing, and insights gathering from the project team in an iterative manner. The other supporting computer programs mentioned previously are considerably simpler, requiring much less programmer effort, but they do add to the fixed costs. Overall, this investment is large enough to be noticeable, but not so large as to keep an institution of higher education from proceeding down this path. In comparison, the cost of developing a MOOC is typically in the $150,000–$250,000 range Online Learning Insights^17^, and yet many institutions have been very willing to make this speculative investment. The investment discussed here is only a fraction of that amount. Using the items in the software repository GitHub^18^ it is likely that <1 week of programmer time would be required with an estimated cost of roughly $2,000. To use this tool in a new environment, the cost will also include the time needed to figure out the format in which the data is obtained from the LMS or other source, if that is not already known. The Concept Table and the quiz metadata must be developed separately for each course. However, the computer programs can be used for all courses without modification, greatly decreasing the cost per course. There is likely to be a need for continued development (e.g., to increase the flexibility for handling partial credit for quiz question answers), but that will still allow the analysis of all courses with that one set of programs.

A major portion of the fixed cost in our methodology arises in providing the metadata required for the analysis to give formative feedback to individual students. Producing, for each quiz, the two types of metadata described previously requires a significant investment in thought and time. This, again, is a fixed cost. It is then applied to all the students in the course, which will usually be a large number, with zero marginal cost. With an additional, but typically considerably lesser, investment in providing revision for subsequent semester usage, it continues to provide economies of scale as the initial cost will also be distributed over the future students.

The marginal cost incurred in providing this support to an increased number of students in a course comes down to the cost of computer time taken to perform the analysis and output the reports. Somewhat surprisingly to many, this marginal cost is essentially zero, using <1 min per test on a desktop computer, even for a large class. Distributing the reports to the students electronically, such as via e-mail, adds insignificant marginal cost, as tools, such as e-mail are considered to have such low marginal cost as to not be metered and billed.

With such low marginal costs, only the fixed “up-front” costs have an impact on the institution's budget. The result is that this approach provides great economies of scale. It should, therefore, be considered first for large (or very large) enrollment courses. However, the ease of adoption and implementation certainly allows its use in smaller enrollment courses.

### Strengths/benefits of the tool

Our method is constructed to assist student learning within existing courses, thereby increasing course completion rates and doing so in an affordable manner. Our approach is consistent with the four principles Wise (Wise, 2014) describes for designing interventions for student use of learning analytics. Our method can be used in conjunction with any course, with any textbook (and any other presentation materials) without altering them (i.e., without use of an authoring system and permission to rework and distribute the textbook and other materials), and without requiring the adoption of a textbook with associated services from a publisher. It does require an investment to develop the metadata for the concepts, resources, and quizzes for a course. This means that there is an up-front effort required, but no resource or equipment costs, and the up-front effort is distributed over the initial use in a course and following courses using the same material. There needs to be an incremental investment to adapt to changes in the course, including when using new instructional source materials or simply updating the course based on experience, but this is significantly lower than in the initial implementation. Our method also works with any LMS, and any student desktop or mobile digital devices.

## Results

The information produced by our programs' analyses of a student's performance can be presented to the student who has a problem with a concept. This provides guidance to the student to review concepts and to make sure that the IPCs are understood. This procedure should be explained to the students at the beginning of the course.

Feedback is tailored to each individual student to assist in making needed progress. It is this additional dimension that can supply some of the individual interaction that is lost as class sizes climb. Our proposal cannot fully replace the individual person-to-person interaction between a student and an instructor, but it can decrease the amount of time that a student requires of an instructor. Our tool gives students guidance about what they can and should study on their own before seeking assistance from an instructor. Our tool offers the added benefit of decreasing the amount of time that a faculty member must spend with an individual student who seeks help, because the computer feedback (Figure 6) can help direct and focus the conversations between the student and instructor. The faculty member does not have to spend significant amounts of time trying to understand and determine the concepts or skills that are challenging the student because our tool, in conjunction with good test items, has already identified that for the instructor. Often, students in large classes are intimidated by the prospect of reaching out for help because they do not know how to begin the conversation and they do not want to overburden an instructor whom they know has many students. The feedback from our tool may help to make faculty contact more approachable for students because it can serve as a starting point for the conversation. When, as is the case in large classes, the person-to-person interaction is limited, our methodology can help supply a portion of what is needed to support student learning.

### Student engagement

Our methodology relies on the student paying attention to the feedback and using it as a guide to study and to engaging with the faculty. Our experience, in the courses that have tested this methodology, is that there is a heterogeneity in the class. Many of the students use the feedback in the desired manner, and often go so far as to thank the instructor. Unfortunately, a significant fraction of the class does not pay attention to the feedback and even won't open the email providing the individual feedback (Figure 6). This email has a Subject: header which clearly identifies the content, and so this appears to be intentional indifference. The instructor's job of motivating the students is not endangered by our methodology, and needs to be emphasized even as our methodology replaces much of the lower level task burden in a large class.

## Conclusions

The missing element in most current efforts aimed at improving student progress and retention is providing support to students on their difficulties in learning the subject matter within a course. The existing attention to engagement, motivation, study habits, etc. is extremely important and should continue to be improved, but it is limited in its effect. Especially for larger courses, instructor interaction with each individual student is very small, if present at all, and so students who miss a concept are left to drift without individual attention. They may have the good fortune of having study partners who supply the missing attention, but, especially for online education, they may not be so fortunate. Supplying individual attention from the instructor or teaching assistant is limited by the cost, which scales essentially linearly with class size, and so is limited during these times of budget stresses.

Our methodology, which uses each test as a diagnostic instrument to provide formative feedback to each student, has a very different cost structure. It has a significant, but affordable, fixed cost. The marginal cost of usage for each additional student in the course is essentially zero. The fixed cost for a course consists of dissecting the material into separate concepts on which the learning outcomes depend, and of delineating the structure of relationships among the IPCs identified for each concept. Additionally, metadata must be added to each test item, noting the concept(s) tested by that question, as well as the condensed Bloom's Cognitive Taxonomy level. With these metadata, the quiz results are analyzed by a computer program (another fixed cost) to provide a report for each student, yielding a concepts-missed profile as well as a Bloom's profile. This formative feedback can then be used by the motivated student for guidance in studying and for improving the chance of satisfactory completion of the course. It also provides feedback and class progress and difficulties to the instructor.

The goal of increasing student retention and decreasing time to graduation depends upon improving satisfactory course completion rates. It is this basic building block which our methodology improves, and it does so in an affordable and scalable manner.

## Ethics statement

The student data in Figures 5 and 6 are fabricated. When pilot testing the software in real classes, test performance of each student was analyzed with no personally identifiable information being exposed other than to the instructors. The Chair of the NC State Univ. Institutional Review Board for the Protection of Human Subjects in Research (IRB) said that this did not require review or approval.

## Author contributions

HS, KY, EL, and DC made substantial contributions to the development of the concepts and the analysis of the data, to the drafting and approval of this manuscript and agree to be held accountable for all aspects of this work. HS originated the project and provided all of the computer programming and computer analyses.

### Conflict of interest statement

The authors declare that the research was conducted in the absence of any commercial or financial relationships that could be construed as a potential conflict of interest.

## Footnotes

^1^Blackboard. Available online at: http://www.blackboard.com (Accessed Nov14, 2016).

^2^Ellucian. Available online at: http://www.ellucian.com (Accessed Nov14, 2016).

^3^Starfish Retention Solutions. Available online at: http://www.starfishsolutions.com (Accessed Nov14, 2016).

^4^GradesFirst. Available online at: http://www.gradesfirst.com (Accessed Nov14, 2016).

^5^Civitas Learning. Available online at: http://www.civitaslearning.com (Accessed Nov14, 2016).

^6^Ellucian. Available online at: http://www.ellucian.com (Accessed Nov14, 2016).

^7^Starfish Retention Solutions. Available online at: http://www.starfishsolutions.com (Accessed Nov14, 2016).

^8^GradesFirst. Available online at: http://www.gradesfirst.com (Accessed Nov14, 2016).

^9^Acrobatiq. Available online at: http://www.acrobatiq.com (Accessed Nov14 2016).

^10^Smart Sparrow. Available online at: http://www.smartsparrow.com (Accessed Nov14, 2016).

^11^WebAssign. Available online at: http://www.webassign.com (Accessed Nov 14, 2016).

^12^Aplia. Available online at: http://www.aplia.com (Accessed Nov 14, 2016).

^13^CengageNow. Available online at: https://www.cengage.com/services/ (Accessed Nov 14, 2016).

^14^Cengage MindTap. Available online at: http://www.cengage.com/mindtap/ (Accessed Nov 14, 2016).

^15^LearnSmart. Available online at: http://www.learnsmartadvantage.com (Accessed Nov 14, 2016).

^16^Wiley. Available online at: http://www.wiley.com/college/sc/oriondemo/ (Accessed Nov 14, 2016).

^17^Online Learning Insights, “How and why institutions are engaging with MOOCs answers in report MOOCs expectations and reality,” Available online at: https://onlinelearninginsights.wordpress.com/tag/costs-of-developing-a-mooc/ (Accessed Nov 14, 2016).

^18^https://GitHub.com/hes8/formassess (Accessed Feb 17, 2017). Archived at https://zenodo.org [We have requested a DOI from Zenodo with no response.]
